# Effect of hyaluronic acid on chondrocyte apoptosis

**DOI:** 10.1590/1413-785220152302144341

**Published:** 2015

**Authors:** Ronald Bispo Barreto, David Sadigursky, Marcia Uchoa de Rezende, Arnaldo José Hernandez

**Affiliations:** 1 Universidade de São Paulo, Faculdade de Medicina, Department of Orthopedics and Traumatology, São Paulo, SP, Brazil, 1. Department of Orthopedics and Traumatology, Faculdade de Medicina da Universidade de São Paulo, São Paulo, SP, Brazil

**Keywords:** Chondrocytes, Hyaluronic acid, Apoptosis, Rabbits, Knee, Osteoarthritis

## Abstract

**OBJECTIVE:**

To determine the percentage of apoptotic cells in a contusion model of osteoarthritis (OA) and to assess whether intra-articular injection of high doses of hyaluronic acid (HA) immediately after trauma reduces chondrocyte apoptosis.

**METHODS:**

Forty knees from adult rabbits were impacted thrice with a 1 kg block released through a 1 meter tall cylinder (29.4 Joules). Subsequently, 2 mL of HA was injected in one knee and 2 mL saline in the contra-lateral knee. Medication were administered twice a week for 30 days, when animals were sacrificed. Specimens were prepared for optical microscopy exam and terminal deoxynucleotidyl transferase end labeling assay (TUNEL).

**RESULTS:**

The apoptosis rate in the contusion model was 68.01% (± 19.73%), a higher rate than previously described. HA significantly reduced the rate of apoptosis to 53.52% (± 18.09) (p <0.001).

**CONCLUSION:**

Intra-articular HA administration started immediately after trauma reduces impact-induced chondrocyte apoptosis rates in rabbits.

**Level of Evidence I, Experimental Study.:**

## INTRODUCTION

Articular cartilage has a limited reparative capacity.^1^ Histological studies have shown the occurrence of chondrocyte death (apoptosis or necrosis) in response to trauma.^1^^-^ 3 Apoptosis was studied in other tissues and cells, and various inducers were identified, including chemical agents, cytokines, viral and bacterial pathogens, and thermal injuries.^4^^,^ 5 This programmed cell death process plays a critical role in embryonic development and homeostasis.^6^ Apoptosis can also be caused by mechanical stress in a variety of cells.^7^^,^ 8

Apoptosis occurs in osteoarthritis chondrocytes and in response to mechanical injury in vitro. It is possible that the appearance of chondrocyte apoptosis is one of the early responses to mechanical injury.^6^^,^ 9 ^-^ 11 An average rate of 35% in chondrocyte death was found in osteochondral fragments removed after intra-articular fractures in humans. This rate is more than double than the rate found in knees with osteoarthritis (15%). High apoptosis rates help explain the occurrence of post-traumatic osteoarthritis, even in intra-articular fractures anatomically fixed.^12^

Prevention of post-traumatic arthrosis is directly related to the prevention of apoptosis.^13^ Many pharmacologic agents can block apoptosis and enhance cell survival. Some of these agents include inhibitors of caspases (cysteine-dependent aspartate specific proteases), glucosamine, diacerein, hyaluronic acid, platelet rich plasma and osteogenic -1 protein (OP-1).^14^^-^ 19 The intra-articular injection (IA) of glucosamine in rabbits played a chondroprotective effect, reducing the degradation of articular cartilage while suppressing synovitis.^15^ In dogs, IA and intravenous (IV) injection of hyaluronic acid (HA) suppressed chondrocytes apoptosis after surgical resection of the anterior cruciate ligament (ACL),^18^ as well as after cartilage damage mediated by fibronectin fragments.^20^

A progressive increase in the number of apoptotic cells starting six hours after the trauma has been noticed, providing a potential therapeutic window in the first six hours after injury.^13^ In rabbits, the immediate and continuous administration of diacerein for three months after injury reduced post-traumatic osteoarthritis, as well as the use of platelet-rich plasma (PRP).^16^ HA can slow the degeneration of articular cartilage and inhibit the production of reactive oxygen species and prostaglandin E2; therefore, the hypothesis raised is that early administration of HA reduces apoptosis of chondrocytes after injury. To test the hypothesis, a contusional model of knee osteoarthritis (OA) was made in rabbits, which measured the apoptosis rate with or without the immediate administration of HA.

The objective of this study was to evaluate whether high doses of HA may reduce or inhibit apoptosis subsequent to a post-contusional model of OA,^17^ and whether the model is low, medium or high energy, since it has not been described for measuring apoptosis.

## METHODS

The study was approved by the local Ethics Committee (CAPPesq 0884/09) and developed at the Laboratory of Medical Research (LIM41), Department of Orthopedics and Traumatology, Faculdade de Medicina da Universidade de São Paulo.

We studied 20 rabbits (40 knees) of the New Zealand breed, with a mean age of eight weeks and weighing 4-6 kg. Each animal received the intervention in one side, while the contralateral side served as control according to a randomization list (www.randomization.com).

Each animal received an intramuscular injection of 40 mg/kg ketamine, associated to 5 mg/kg xylazine hydrochloride in the proximal region of the hindlimb.

The contusional model described by Mazières was reproduced three times (29.4 Joules) in each knee with the animals in the supine position.^17^ Blunt trauma was applied to the patella and medial femoralcondyle by the impact of a cylindrical metal weight of (4 cm diameter) released through a 1 m long cylinder (internal diameter 4.1 cm).

After injury, animals received enrofloxacin for seven days (10 mg/kg every 12h subcutaneously), meloxicam (0.1 mg/kg every 24 hours subcutaneously) for four days and tramadol hydrochloride (4 mg/kg subcutaneously every 12 hours) for three days. The animals were kept in individual cages.

The control knees were injected with 2 ml of saline, and knees which underwent intervention were injected with 2 ml (20 mg) HA (Polireumin(r)/Hyalgan(r), TRB Pharma - Brazil). HA or saline was injected immediately after injury and 3, 7, 10, 14, 17 and 21 days post-injury. The injections consisted of intramuscular injection of ketamine (35 mg/kg) and midazolam (1 mg/kg) and propofol (4 mL). Animals were weighed twice a week before undergoing anesthesia.

**Figure 1.**
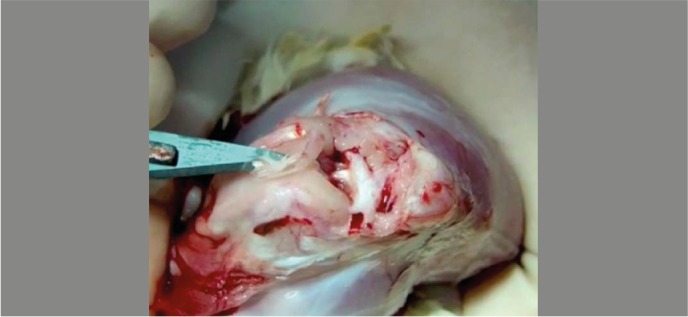
Extraction of cartilage. In detail, the right knee is flexed 90° with the patella displaced laterally. Cartilage of the trochlea and lateral femoral condyle being dissected by the scalpel blade

Thirty days after injury, all animals were sacrificed by intravenous administration of 75 mg/kg thiopental sodium. Immediately after euthanasia, the trochlea and the medial condylar cartilages were excised through a para-patellar medial incision without damaging the subchondral bone. (Figure 1)

The samples were fixed in formalin and embedded in paraffin for histopathological examination in the Anatomic Pathology laboratory of Hospital das Clínicas da Universidade Federal da Bahia. Five millimeters thick sections were prepared on silanized slides. The slides were stained with hematoxylin and eosin (HE) to assess the section condition and were stained using the ApopTag peroxidase in situ Apoptosis Detection Kit (USA) (deoxynucleotidyl terminal transferase, TUNEL).

**Figure 2.**
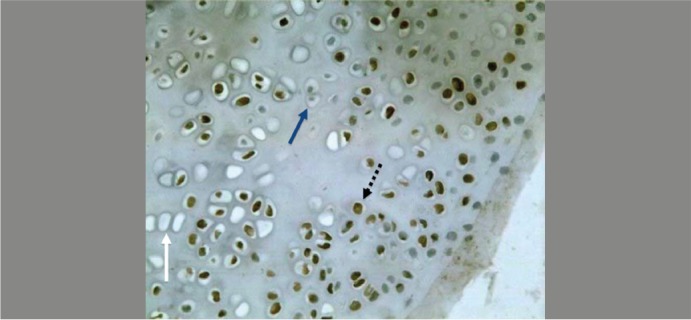
Micrograph of the histological session prepared using the TUNEL technique (400x magnification), showing the chondrocyte nuclei stained in dark blue (viable cells, black arrow), brown stained nuclei (apoptotic nuclei, dotted arrow) and chondroblasts (empty spaces, white arrow).

Brown stained nuclei indicated apoptosis, while the blue nuclei indicated viable cells. (Figure 2) All sections cells stained present in the section, excluding the areas with artifacts were manually counted.

This experimental model generated an impact of about 9.8 J (1 kg dropped from a 1m height). Each animal received three times the impact as previously described.^17^ Energy levels of the impacts and cell death results from this work and previous studies were compared to contusional models in rabbits.^12^^,^ 16 21

Estimate of sample N 

Assuming a power of 80%, an alpha error of 5%, a standard deviation rate of 15 for each group and a mean difference of 20%, it was calculated a sample size of 10 subjects per group. Due to various anesthetic procedures, the sample size was doubled.

### Statistical Analysis

Statistical analysis was performed using the Statistical Package for Social Sciences software (SPSS) version 15 and Microsoft Excel 2003. The null hypothesis (H0) was as follows: the mean difference of the percentage of apoptosis between treated and untreated knees (control knees) is equal to zero. The measurements were compared by the Student t-test setting 5% significance level. The Pearson correlation coefficient was used to evaluate the correlation between the different variables.

## RESULTS

All animals recovered well from multiple anesthetic procedures. Two rabbits died during the experiment due to unknown causes. They progressively lost weight during the study period, with no significant signs of infection or allergic reaction. The remaining 18 were analyzed, and their weight increased significantly during the study (p=0.023). (Table 1)

**Table 1.** Initial and final weight, percentage of apoptosis and results of comparative tests.

	Mean	SD	Median	Minimum	Maximum	N*	p
Weight – initial (g)	4878.61	421.45	4875	4250	6100	18	0.023
Weight - final (g)	5043.33	399.55	5000	4450	6120	18	
% apoptosis Control Group	68.01	19.73	67.05	18.50	95	18	< 0.001
% apoptosis HA Group	53.52	18.09	54.10	5.50	80.50	18	

SD: Standard deviation; *Number of knees.

**Figure 3.**
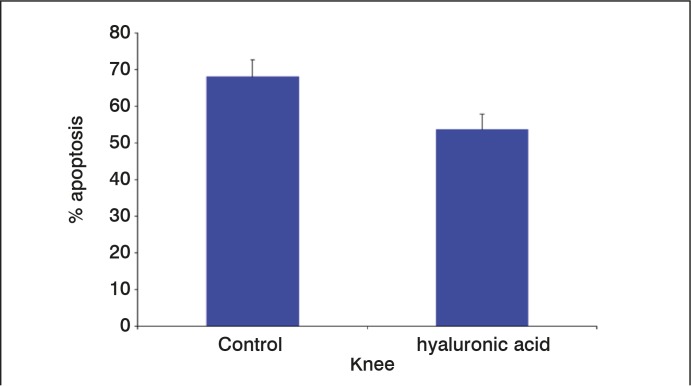
Mean percentage of apoptosis in each knee with respective standard errors.

There was a high variation in the absolute number of cells in each knee, which led us to determine the percentage of apoptose.^12^ We observed that apoptosis in the knees that received hyaluronic acid (Figure 3) was lower than the control knees (p <0.001). (Table 1) The difference in percentage of apoptosis between control knees and knees injected with HA did not correlate with changes in weight or the total number of cells (Pearson correlation coefficient, Table 2).

**Table 2.** Pearson correlation coefficient between differences in apoptosis percentages and alterations in weight and total number of analyzed cells.

	Correlation	N*	p
Weight change	0.091	18	0.448
Total number of cells	-0.093	18	0.713

*Number of knees.

Table 3 shows the comparison of results obtained in this study with the results of a less aggressive contusional model of OA.^21^ The impact energy in this study was much higher, as well as a significantly higher rate of cell death.

**Table 3.** Comparison of different levels of energy and cell death in different contusion models of osteoarthritis in rabbits.

	Mazières	Rundell
Trauma Energy (J)	29.4	6
Apoptosis (%)	68.01	31%

## DISCUSSION

Prevention of post-traumatic arthrosis is directly related to the prevention of apoptosis.^13^ The impacted chondrocytes die by necrosis (in the first 12h after impact) or after, by activation of a specific metabolic cascade that triggers apoptosis;^22^ this suggests the presence of a therapeutic window in which the process could be inhibited. Thus, apoptosis keeps the process of cell death in response to the initial impact. The pharmacological inhibition of caspases was shown to reduce the seriousness of cartilage injuries.^14^ A study has been proposed to determine the number of apoptotic cells in contusional OA model described above and to test and the effect of intra-articular administration of HA on chondrocytes apoptosis induced by high energy trauma.^17^

A limitation of our study is the use of only one technique for assessing cell death. The programmed cell death in human cartilage can be analyzed by using one of four independent methods (terminal deoxynucleotidyl transferase, TUNEL; analysis of DNA denaturation using a specific antibody for single stranded DNA, ssDNA; immunohistochemical detection of caspase-3; and in situ oligonucleotide binding). Each of these methods detects different steps along the apoptotic process.^23^ We chose the TUNEL method because it can identify cellular changes in the early stages of osteoarthritis, before the physical changes are visible. This makes it the most attractive technique in pharmacological studies in animal models^9^ and for comparison with previous published results.^18^ Another limitation of our study is that the control group received saline injection twice a week, what could reduce the occurrence of apoptosis. Thus, the average rate of 68.01% apoptotic cells in this study varying from 18.5% to 95% (Table 1), could have been even higher if no intra-articular injection of saline would have been made. However, the model is effective in inducing OA, due to its high apoptosis rate.

In general, the previously described models for studying apoptosis use mechanical or chemical means to produce cartilage injuries. The mechanical trauma reproduces events that lead to post-traumatic OA. Mechanical models, such as those which surgically section the bilateral^14^ or unilateral^15^ anterior cruciate ligament (ACL) do not reproduce the actual trauma involved in ACL rupture, because there is no disruption of the capsule or the collateral ligaments, nor meniscal injuries or bone and cartilage contusions are present in ACL ruptures observed clinically, in which the energy involved in knee dislocation is greater. However, the unstable knee produced by ACL surgical section causes recurring micro-trauma of the cartilage, thereby, eventually causing OA. Possible contusional models include direct damage to the cartilage through a small arthrotomy, a contusion articular injury,^17^ and intra-articular fracture of the medial femoral condyle. We chose Mazières' contusional model for three reasons: 1) The immediate apoptosis-inducing ability; 2) Higher energy involved, similar to what occurs in closed intra-articular fractures and bone contusions; and 3) Lack of information on cell death for this contusional model in the literature.^17^

Apoptosis rates observed were high, as the applied energy. On average, we observed 68.01% of apoptotic cells (range 18.5 to 95%). (Table 1) These values ​​are higher than those found in intra-articular fractures in humans.^12^ In the literature, transection of the ACL caused 28.7% apoptosis in rabbits,^8^ and 24.5% in dogs.^18 ^A punctual impact of 6 J in rabbit patella caused 31% apoptosis (Table 3).^21^ In intact explants of bovine cartilage submitted to a single injury of 500-ms load of 30% of a radially unconfined compressive deformation, apoptosis reached 43%.^1^ The differences between the results in both studies can be assigned to different energies used. There is a direct dose response relationship between the load applied to the cartilage and chondrocyte apoptosis rate.^1^ In our study, the kinetic energy accumulated at the moment in which the weight reaches the patella was about 9.8 J at each impact; and it was repeated three times on each knee to provide a total of about 30 J. Thus, the energy of a single trauma in our study was 63% higher than that performed by Rundell *et al*.,^21^ which was of 6 J (1.3 kg dropped from a height of 46 cm), causing significant morphological changes in cartilage and 31% chondrocyte apoptosis. Therefore, it is believed that the high energy trauma applied in this experiment is responsible for the high rate of cell death.^21^

The decision to use hyaluronic acid was based on the accessibility and properties of the drug; it was also an attempt to bring the experimental method into clinical practice, which was the ultimate goal of this study. To verify whether the therapeutic effects were due to HA and not to the saline solution vehicle, one of the two knee of each rabbit received HA, while the other received only the vehicle (0.9% saline). Various types of HA are described - HA having molecular weights of 0.12 to 0.6×10^6^ Da, 0.8×10^6^ Da and 1.2×10^6^ Da. HA with a molecular weight (MW) of 0.5 -0.75×10^6^ is considered low, whereas 1×10^6^ Da HA is considered high. The interactions of these polymers are complex and it is not possible to determine what type of HA has better rheological behavior. Low MW HA preparations show a higher ability to penetrate the synovial membrane in the extracellular matrix (ECM), reducing synovitis and restoring the rheological properties of synovial fluid. Agents with higher MW offer more analgesic power and stimulate less the production of nitric oxide (NO).^24^ It was decided to use the low-MW HA, Polireumin(r)/Hyalgan(r) (TRB-Pharma, Switzerland), which has a molecular weight in the range 0.5 to 0.75 × 10^6^ Da, due to its greater ability to penetrate the ECM and because it has been used in numerous studies in animals.

In the present study, about 7 mg/kg/dose in a total of seven doses was administered. Usually, a dosage of 1 mg/kg/week is used at. The reasons for using such a high dose were as follows: to ensure a constant intra-articular HA concentration, since the free radicals produced in inflamed joints cause oxidative damage to the HA and diminish its concentration in synovial fluid. It was also hypothesized that best results would be achieved by performing the intervention during the acute phase of trauma (the first three to four weeks) and, therefore, we prolonged the treatment to seven doses (in a 21 days period). This hypothesis was based on a pilot study (unpublished) developed by our group, where the same experiment was conducted using three doses (within 15 days) instead of seven doses. No significant differences were found in the pilot study.

Injection of knees with HA inhibited apoptosis in chondrocytes, i.e., our results support the hypothesis that hyaluronic acid can help to preserve the chondrocytes after mechanical injury to the articular cartilage. (Table 1) There was a 14.49% reduction in the percentage of cells undergoing apoptosis, due to the effect of HA in our experimental model. This result is comparable to Rundell's *et al*.^21^, who observed a decline in cell death rate from 31% in controls to 16% in knees treated with surfactant poloxamer 188 (P188). We found similar reductions to those described by Echigo *et al*.,^18^ who administered HA on dogs knees after ACL section. These authors observed a decrease in apoptosis rate from 24.5% (control) to 12.7% (treated group). Together with data from previous studies, this study supports the use of pharmacological agents to preserve the viability of chondrocytes after traumatic injury.^17^

Although we found a decrease in apoptosis rates with HA, it is known that the residual rate of 53.52% would be sufficient to generate post-traumatic osteoarthritis.^12^ Although Martin *et al*.^22^ have also found a high proportion of dead cells (36% in the group treated with N-acetylcysteine), our rate is higher than most of the published rates.

In summary, we found a decrease in apoptosis rates using HA. However, apoptosis levels remained high in this contusional model. Possibly, in lower energy trauma HA can reduce these rates to lower levels than those required to cause osteoarthritis.

## CONCLUSION

After contusional injury on rabbits' knees the immediate administration of HA and for subsequent 21 days reduced apoptosis rates.
